# RSU-1 Maintains Integrity of *Caenorhabditis elegans* Vulval Muscles by Regulating α-Actinin

**DOI:** 10.1534/g3.120.401185

**Published:** 2020-05-27

**Authors:** Xinyan Wang, Shuai Huang, Cunni Zheng, Wei Ge, Chuanyue Wu, Yu Chung Tse

**Affiliations:** * Department of Biology,; ^§^ Guangdong Provincial Key Laboratory of Cell Microenvironment and Disease Research, Shenzhen Key Laboratory of Cell Microenvironment, Southern University of Science and Technology, Shenzhen, 518055, China,; ^†^ Centre of Reproduction, Development and Aging, Faculty of Health Sciences, University of Macau, China, and; ^‡^ Department of Pathology, University of Pittsburgh School of Medicine, PA 15261

**Keywords:** α-actinin, Egg-laying, Ras, RSU-1, Vulval muscle cells

## Abstract

Egg-laying behavior in *Caenorhabditis elegans* is a well-known model for investigating fundamental cellular processes. In egg-laying, muscle contraction is the relaxation of the vulval muscle to extrude eggs from the vulva. Unlike skeletal muscle, vulval muscle lacks visible striations of the sarcomere. Therefore, vulval muscle must counteract the mechanical stress, caused by egg extrusion and body movement, from inducing cell-shape distortion by maintaining its cytoskeletal integrity. However, the underlying mechanisms that regulate the cellular integrity in vulval muscles remain unclear. Here, we demonstrate that *C. elegans* egg-laying requires proper vulval muscle 1 (vm1), in which the actin bundle organization of vm1 muscles is regulated by Ras suppressor protein 1 (RSU-1). In the loss of RSU-1, as well as Ras^LET-60^ overactivation, blister-like membrane protrusions and disorganized actin bundles were observed in the vm1 muscles. Moreover, Ras^LET-60^ depletion diminished the defected actin-bundles in *rsu-1* mutant. These results reveal the genetic interaction of RSU-1 and Ras^LET-60^ *in vivo*. In addition, our results further demonstrated that the fifth to seventh leucine-rich region of RSU-1 is required to promote actin-bundling protein, α-actinin, for actin bundle stabilization in the vm1 muscles. This expands our understanding of the molecular mechanisms of actin bundle organization in a specialized smooth muscle.

Hermaphrodite *Caenorhabditis elegans* fertilize their oocytes with their own sperms and stores 10 to 15 fertilized eggs in the uterus, after which the eggs are expelled into the environment through the vulva in every 20 min (Waggoner *et al.* 1998). Although this egg-laying behavior has been studied intensively to understand the function and development of the nervous system (Schafer 2006), studying the development of vulval muscle cells also helps in elucidating diverse fundamental cellular mechanisms, including cell migration, adhesion, and signal transduction (Bastiani *et al.* 2003).

Animals harbor two types of muscles – striated somatic muscle and nonstriated smooth muscle. Whereas somatic muscle consists of multiple sarcomeres, which are functional contractile units featuring a specialized organization of the M-line, dense body (Z-disk), actin filaments, and myosin filaments (Moerman and Williams 2006), smooth muscle lacks these highly organized sarcomeres. In *C. elegans*, body-wall muscle is striated muscle (Gieseler *et al.* 2018), while pharyngeal, vulval, uterine, anal, and intestinal muscle are nonstriated smooth muscle. Proper egg-laying in *C. elegans* requires smooth muscle contraction and relaxation. Vulval muscle cells function in regulating the opening of the vulva. Two types of vulval muscle cells have been identified in *C. elegans*, vm1 and vm2. The four vm2 vulval muscles arearranged in an X-shaped pattern, with their apical ends attaching to the vulva, and the cells receive synaptic input from hermaphrodite-specific neurons (HSNs) and ventral cord type C neurons (VCs). Ablation of vm2s eliminates egg-laying (White *et al.* 1986). Conversely, the four vm1s display a similar pattern as vm2s and are electrically coupled to vm2s, but the vm1s do not receive any synaptic input (White *et al.* 1986). Therefore, vm1s might facilitate the vulval opening process in a neuronal circuit-independent manner.

The proteins that localize at the dense body of sarcomeres are equivalent to the proteins in the integrin-mediated cell-adhesion complex. Integrins, which are transmembrane heterodimeric receptors highly conserved from nematodes to mammals, facilitate the mechanical linkage of the extracellular matrix to the cytoskeleton (Brown 2000; Hynes 2002; Huveneers *et al.* 2008). Integrin-mediated signaling pathways regulate several cellular processes, including cell adhesion, migration, proliferation, and differentiation (Schwartz and Ginsberg 2002; Huveneers *et al.* 2007; Humphries *et al.* 2019). Integrins (*e.g.*, PAT-2 and PAT-3 in *C. elegans*) consist of α and β subunits, each containing a large extracellular domain, a single transmembrane domain, and a short cytoplasmic tail. Once activated to the extended-open conformation, Integrin^PAT-3^ recruits integrin-linked kinase (ILK^PAT-4^), particularly interesting Cys-His-rich protein (PINCH^UNC-97^), and parvin^PAT-6^ to form the IPP (Integrin-PINCH-Parvin) complex (Tu *et al.* 1999; Zhang *et al.* 2002; Wu 2004; Legate *et al.* 2006; Wickström *et al.* 2010). The IPP complex regulates cell adhesion, F-actin polarization, vascular smooth muscle contractility, and microtubule organization and dynamics (Wu 2004; Legate *et al.* 2006). Loss of Integrin^PAT-3^, ILK^PAT-4^, and PINCH^UNC-97^ in *C. elegans* causes Pat (Paralyzed and Arrested elongation at Twofold) and Unc (uncoordinated) phenotypes (Williams and Waterston 1994; Gettner *et al.* 1995; Mackinnon *et al.* 2002), which are related to the dysfunction of muscular tissues.

Besides the key components of the IPP complex, RSU-1, a Ras suppressor, localizes at focal adhesion sites. RSU-1 coimmunoprecipitates with the PINCH-ILK complex through its interaction with the LIM5 domain of PINCH^UNC-97^ (Kadrmas *et al.* 2004). RSU-1 is a 33-kD protein with 7 leucine-rich repeat (LRR)-containing domains. Human cell lines lacking RSU-1 are viable, but the proliferation and migration of the cells are markedly compromised (Gonzalez-Nieves *et al.* 2013); conversely, the overexpression of RSU-1 disrupts actin-filament networks (Masuelli and Cutler 1996). Moreover, RSU-1 might function in the absence of PINCH-ILK functions. In *Drosophila* exhibiting PINCH-ILK interaction deficiency, RSU-1 is essential for viability (Elias *et al.* 2012); RSU-1 participates in regulating the p38 MAP kinase signaling pathway (Gonzalez-Nieves *et al.* 2013); and RSU-1 promotes the localization of extrasynaptic acetylcholine receptors (AChRs) at the neuromuscular junctions (NMJs) of dorsal and ventral SAB motor neurons (Pierron *et al.* 2016). These findings indicate that RSU-1 potentially participates in multiple cellular processes, and the underlying molecular mechanisms on how RSU-1 suppress Ras is largely unclear.

Here, we report that RSU-1 functions in concert with Ras^LET-60^ to regulate actin bundling in the vulval muscle cells of *C. elegans*. We demonstrated that egg-laying ability was substantially reduced in a *rsu-1* null mutant and a Ras^LET-60^ overactivated mutant. By performing genetic investigations and live imaging of *C. elegans* vulval muscle cells, we further demonstrated that RSU-1 and proper levels of Ras^LET-60^ are required for maintaining normal cellular structure and actin bundling in vm1 vulval muscles; the data from our genetic analyses also indicated that RSU-1 inhibits the activity of Ras^LET-60^. In addition, by rescue experiments, we demonstrated that the fifth to seventh leucine-rich domain of RSU-1 is required to regulate Ras^LET-60^. Lastly, we showed that α-actinin (ATN-1) acts as a downstream effector in RSU-1-mediated signaling to promote F-actin bundling. Based on our findings, we propose that RSU-1 inhibits Ras^LET-60^ activity to regulate proper actin bundling in *C. elegans* vulval muscle.

## Materials And Methods

### C. elegans strains

All nematode strains were cultured at 22° on NGM (nematode growth medium) plates seeded with *Escherichia coli* OP50. All strains used in this study are listed in Table S1.

### RNAi

RNAi experiments were performed using the feeding method (Timmons and Fire 1998). We obtained *let-60**(RNAi)* clones from Julie Ahringer’s library (Kamath and Ahringer 2003), and we constructed *rsu-1**(RNAi)* and *atn-1**(RNAi)* by inserting the corresponding DNA fragments into L4440 vectors. RNAi plasmids were transformed into bacteria HT115. Primers used for DNA amplification are listed in Table S2. RNAi feeding plates were prepared using NGM containing 100 μg/ml ampicillin and 1 mM isopropyl-β-thiogalactoside. HT115 carrying RNAi plasmids were grown in 5 ml of LB containing 100 μg/ml ampicillin at 37° overnight and then seeded on the RNAi plates at room temperature for 10 h. Embryos were hatched on the RNAi plates, and then at the L4 stage, were transferred to new RNAi plates for 12 h and used for confocal imaging.

### Egg-laying assays

The average numbers of laid eggs were quantified as described (Trent *et al.* 1983): N2 worms in L4 stage—30 each from control and *rsu-1**(**tm6690**)* strains—were treated with 100 μl of serotonin (3 mg/ml) for 60 min in each condition, and then the eggs expelled from each worm were counted. The developmental stages of the eggs were examined using confocal imaging. Whole-worm images were acquired using a Nikon A1R confocal microscope equipped with a 40×/1.4 numerical aperture (NA) objective. Images were processed using ImageJ.

### Fluorescence imaging

Young adult *C. elegans* hermaphrodites were selected for fluorescence imaging; the worms were anesthetized with 0.5% tetramisole on a 5%-agarose pad placed on a glass slide and then were turned over to the ventrolateral position. GFP was visualized using a 488-nm laser, whereas mKate and mCherry were visualized using a 561-nm laser. To obtain images of vulval muscle and the actin filaments in vulval muscle, Z-stack images were acquired using either a Nikon A1R confocal microscope equipped with a 100×/1.4 NA oil-immersion lens and photomultiplier tube (PMT) detector, or an Olympus IX83 spinning-disk confocal microscope equipped with a 60×/1.4 NA objective and a charge-coupled device (CCD) camera. Images were processed using ImageJ.

### Phalloidin staining

To visualize actin filaments in vulval muscle, young adult worms were stained with iFlour 555-conjugated phalloidin (Shanghai YEASEN Biotechnology; CAT: 40737ES75). Worms were fixed with 3% formaldehyde for 3 h at room temperature, washed thrice with phosphate-buffered saline (PBS), and treated with 100% acetone at -20° for 5 min. Next, the worms were washed with PBS and stained with iFlour 555-phalloidin (0.1 μg/ml) overnight at 4° in the dark, and after rinsing with PBS, the stained worms were examined using a Nikon A1R confocal microscope equipped with a 100×/1.4 NA objective.

### TEM analysis

Worms were washed with M9 buffer, fixed overnight with the primary fixative solution (0.5% glutaraldehyde and 1.5% paraformaldehyde), and then incubated with the secondary solution (2% osmium tetroxide) for 4 h at room temperature. After washing with distilled water, the samples were dehydrated using 25%, 50%, 75%, 90%, and 100% acetone, infiltrated with spur resin at 25%, 50%, 75%, and 100%, and then embedded in a plastic capsule at 60° for 2 days. Samples were sectioned at 60-nm thickness, and the thin sections were treated with uranyl acetate and lead citrate before examination by using a Hitachi HT-7700 TEM instrument.

### Transgenic strain construction

The transgenic strains mCherry::RSU-1 and GFP::PINCH^UNC-97^ were generated by SunyBiotech (China) by using a modified CRISPR/Cas9 method (Dickinson *et al.* 2015). Transgenic strains with vm1 muscle cell-specific expression of LifeAct::mKate and α-actinin^ATN-1^::GFP were also generated by SunyBiotech.

The extrachromosomal array of truncated RSU-1(LRR 1-7), RSU-1(LRR 1-4), RSU-1(LRR 1-5), RSU-1(LRR 1-6), and RSU-1(LRR 5-7) were constructed by amplifying the corresponding DNA fragments from N2 genomic DNA and cloned into modified pCFJ90. Then, the constructs (80 ng/μl) were injected into young adults gonads together with the original marker pCFJ90 (1.25 ng/μl). The expression of extrachromosomal array were then screened under fluorescent microscope.

### Image analysis and quantification

To examine actin filaments in vulval muscle cells (Figure 4A, Figure 5B & E), Z-stack images (0.6-μm intervals) were acquired on a Nikon A1R confocal microscope equipped with a 100×/1.4 NA objective. The images in different focal planes were projected (maximum projection) for further analysis in ImageJ. The midline of actin bundles was determined using the “freehand section tool” in ImageJ.

For fluorescence-intensity analysis, images of mCherry::RSU-1, GFP::PINCH^UNC-97^, and Integrin^PAT-3^::GFP were obtained using a spinning-disk confocal microscope equipped with a 60×/1.4 NA objective (Figure 2D). The intensities of the cytoplasm, focal adhesion patches, and adhesion sites were analyzed using ImageJ, and the C/F ratios were calculated using this formula: C/F = (Intensity_cytoplasmic_ - Intensity_background_)/(Intensity_focal adhesion_ - Intensity_background_). The intensities measured in different groups were compared using two-tailed *t*-tests in GraphPad Prism.

To analyze α-actinin^ATN-1^::GFP patches in vulval muscle (Figure 5D & E), Z-stack images of α-actinin^ATN-1^::GFP were again acquired using the spinning-disk confocal microscope equipped with the 60×/1.4 NA objective. The images in different focal planes were projected (maximum projection) for further analysis in ImageJ. The areas of α-actinin^ATN-1^::GFP patches were measured using the “analyze particles tool” in ImageJ. Data from different groups were compared using two-tailed *t*-tests in GraphPad Prism.

### Data availability

All strains used in this work are available upon request or at the Caenorhabditis Genetics Center (CGC). All data to support the conclusions of this article are present in figures and tables. Supplemental material available at figshare: https://doi.org/10.25387/g3.12319226.

## Results

### Loss of RSU-1 reduces egg-laying

**Figure 1:**
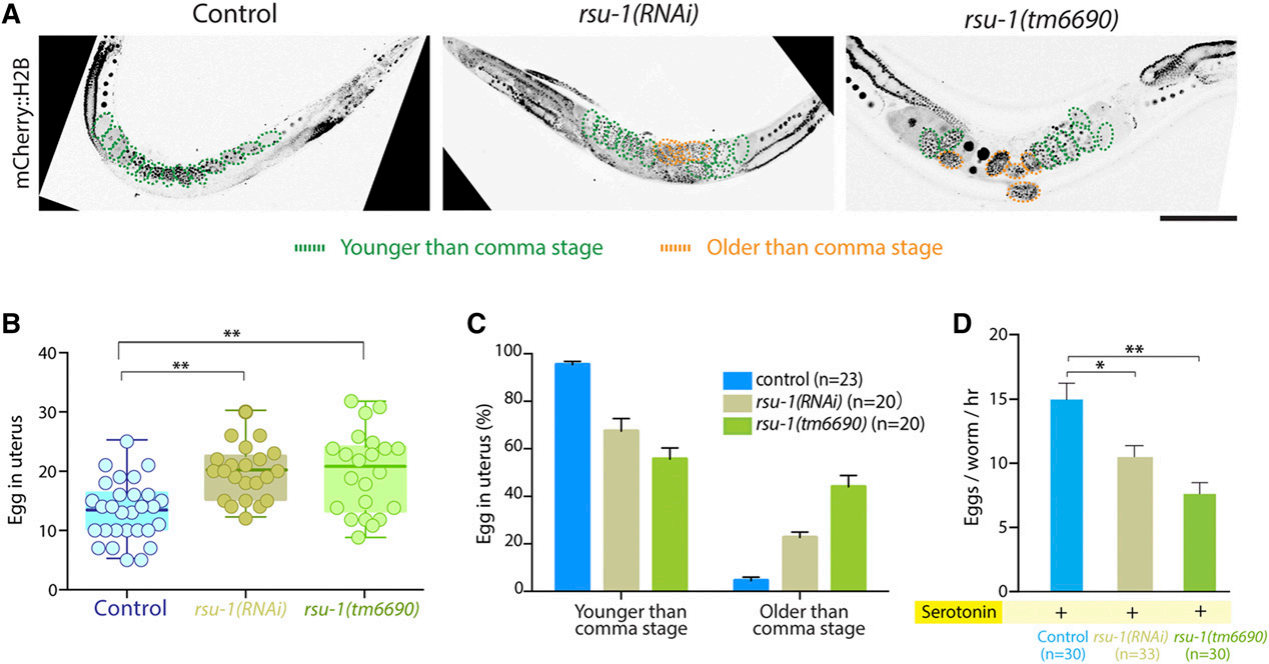
RSU-1 regulates egg-laying. (A) Representative confocal images of mCherry::H2B-expressing adult worms: control, *rsu-1**(RNAi)*, and *rsu-1**(**tm6690**)*. Scale bar, 50 μm. (B) Number of embryos in the uterus of control, *rsu-1**(RNAi)*, and *rsu-1**(**tm6690**)*. (C) Percentages of embryos in different stages in control, *rsu-1**(RNAi)*, and *rsu-1**(**tm6690**)*. (D) Comparison of egg-laying: control, *rsu-1**(RNAi)*, and *rsu-1**(**tm6690*). Data were analyzed using Student’s *t*-test; *P < 0.05, **P < 0.01.

We investigated the role of RSU-1 in vulval muscles by examining the egg-laying ability in *rsu-1**(RNAi)* and *rsu-1**(**tm6690**)* adult worms. The *rsu-1**(**tm6690**)* mutant allele in the chromosome III. We believe that *rsu-1**(**tm6690**)* completely eliminates gene function because it removes the 778 bp from the 5ʹ-end of the locus (Fig. S1). First, we counted the number of unlaid eggs in control, *rsu-1**(RNAi)*, and *rsu-1**(**tm6690**)* adult worms expressing mCherry::H2B (Figure 1A). In control worms, the uterus contained 13 ± 1 fertilized eggs (Figure 1B), which agrees with previous findings (Brewer *et al.* 2019), whereas in the RNAi or mutant worms, the number of unlaid eggs was increased to 18 ± 2 (Figure 1B). Then, we analyzed the developmental stages of unlaid eggs. We observed that the proportion of eggs in the post-comma stage was higher in *rsu-1**(RNAi)* (23% ± 1%) or *rsu-1**(**tm6690**)* (45% ± 2%) than control (5% ± 1%) worms (Figure 1C).

Next, we wonder whether the egg-laying circuits is compromised in *rsu-1**(RNAi)* and *rsu-1* mutant worms. Therefore, we stimulated adult worms to lay eggs by applying the monoamine neurotransmitter serotonin. If RSU-1 acts in the egg-laying circuits, similar amount of laid-eggs will be obtained in the control, *rsu-1**(RNAi)*, and *rsu-1**(**tm6690**)* adult worms after treating with serotonin. Otherwise, less embryos will be expelled in *rsu-1**(RNAi)* or *rsu-1**(**tm6690**)*. In control adult worms treated with serotonin, 15 ± 2 eggs were expelled in 1 h. However, this rate decreased to 11 ± 1 egg in *rsu-1**(RNAi)* and it was almost halved in *rsu-1**(**tm6690**)*, only obtained 8 ± 2 eggs per hour (Figure 1D). These results demonstrated that even the egg-laying circuit was stimulated by serotonin, the number of eggs laid in *rsu-1**(RNAi)* and *rsu-1**(**tm6690**)* adult worms was less than control worms. Collectively, these data suggest that the egg-laying is impaired in *rsu-1** null* mutant worms.

### RSU-1 expresses in vm1 muscles

**Figure 2:**
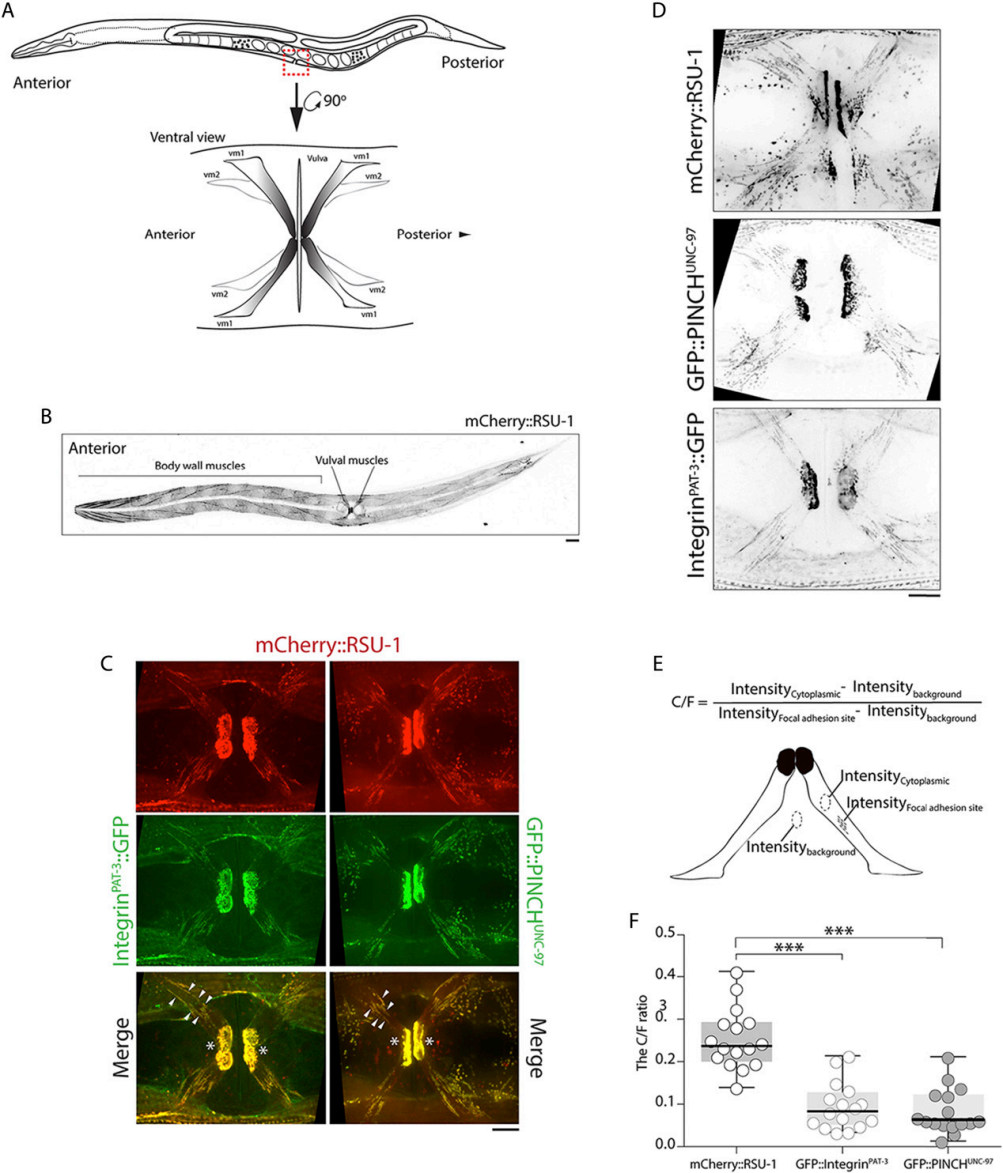
RSU-1 localizes to focal adhesion sites and is diffuse throughout the cytoplasm of vm1 muscles. (A) Vulval muscles in egg-laying system: Schematic showing ventral view of vulval muscle 1 (vm1, gray) and vulval muscle 2 (vm2, white). (B) Representative confocal image of mCherry::RSU-1 localization at body-wall muscle and vulval muscle. Scale bar, 20 μm. (C) Representative confocal images of vm1 muscles expressing mCherry::RSU-1;Integrin^PAT-3^::GFP and mCherry::RSU-1;GFP::PINCH^UNC-97^. Arrowheads: colocalization sites. Scale bar, 10 μm. (D) Representative confocal images of vm1 muscles expressing mCherry::RSU-1, GFP::PINCH^UNC-97^, and Integrin^PAT-3^::GFP GFP::PINCH^UNC-97^. Scale bar, 10 μm. (E) Schematic showing calculation of C/F ratio in vm1 muscles. (F) C/F ratio of mCherry::RSU-1, Integrin^PAT-3^::GFP, and GFP::PINCH^UNC-97^. Data were analyzed using Student’s *t*-test; **P < 0.01, ***P < 0.001.

Considering our finding that RSU-1 contributes to the egg-laying behavior, we further suspected that RSU-1 acts in the vulval muscles, and thereby regulates the vulval opening. First, the subcellular localization of RSU-1 in vulval muscles was examined. We generated a transgenic fluorescent *C. elegans* strain by using the CRISPR/Cas9 genome-editing technique to insert the mCherry-coding DNA sequence into the 5ʹ-end of endogenous *rsu-1* on Chromosome III. Whole-worm imaging revealed that mCherry::RSU-1 was primarily expressed in body-wall muscles and vulval muscles (Figure 2A and B). Then, the subcellular localization of RSU-1 in vulval muscles was further examined by high resolution confocal imaging. We observed that mCherry::RSU-1 were expressed in vm1 muscles. They were primarily accumulated around the vulva, forming large patch in each vm1 muscles (Figure 2C, asterisks), and localized as small puncta throughout the vm1 muscles (Figure 2C, arrowheads). These mCherry::RSU-1-labeled patches and puncta were colocalized with Integrin^PAT-3^::GFP, and GFP::PINCH^UNC-97^ (Figure 2C).

We also noted that mCherry::RSU-1 appeared to have a higher cytosolic fluorescence signal in vm1 muscles as compared to Integrin^PAT-3^::GFP and GFP::PINCH^UNC-97^ (Figure 2D). Therefore, we speculated that an alternative pool of RSU-1 might be diffusely distributed throughout the cytosol in vm1 muscles. To assess this, we measured the average fluorescence intensity in the cytosol and at adhesion sites in vm1 muscles, and then calculated the cytosol-to-adhesion-site intensity ratio (Intensity_cytoplasmic_ - Intensity_background_)/(Intensity_focal adhesion_ - Intensity_background_; C/F ratio) (Figure 2E). The C/F ratios in vm1 muscles expressing mCherry::RSU-1 was significantly higher than that in Integrin^PAT-3^::GFP and GFP::PINCH^UNC-97^ (Figure 2F). Altogether, these results suggest that majority of RSU-1 localizes to the focal adhesion sites, while some of them are present in the cytosol of vm1 muscles.

### RSU-1 regulates Ras^LET-60^ to maintain the cellular structure of vm1 muscles

**Figure 3:**
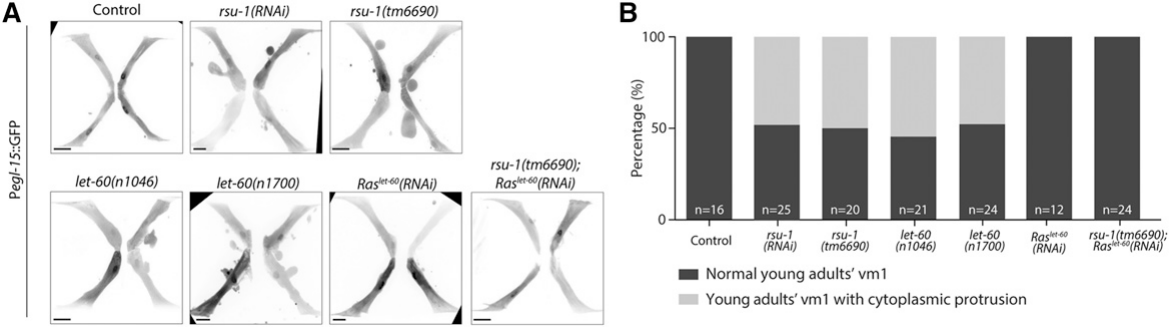
RSU-1 maintains the cellular structure of vm1 muscles through Ras^LET-60^. (A) Representative confocal images of P*egl-15*::GFP, P*egl-15*::GFP;*rsu-1**(RNAi)*, P*egl-15*::GFP;*rsu-1**(tm6690)*, P*egl-15*::GFP;*let-60**(**n1046**)*, and P*egl-15*::GFP;*let-60**(**n1700**)*, P*egl-15*::GFP;Ras^*let-60*^*(RNAi)*, and P*egl-15*::GFP;*rsu-1**(tm6690)*;Ras^*let-60*^*(RNAi)*. Scale bar, 10 and 5 μm. (B) Percentage of vulval muscle showing cytoplasmic protrusions in control, *rsu-1**(**tm6690**)*, *let-60**(**n1046**)*, *let-60**(**n1700**)*, *Ras**^let-60^**(RNAi)*, and *rsu-1**(**tm6690**);Ras**^let-60^**(RNAi)*.

Given that RSU-1 expresses in vm1 muscles, we further investigate the function of RSU-1 by examining the morphology of vm1 muscles in control, *rsu-1**(RNAi)*, and *rsu-1**(**tm6690**)* worms. When cytosolic GFP expression is driven by the vm1 muscle-specific *egl-15* promoter (P*egl-15*::GFP), the morphology of vm1 muscles can be visualized by using confocal imaging. In controls, the 4 vm1 muscles were rod-like in shape and displayed straight and smooth cell boundaries, and cells were oriented toward each other around the vulva and formed a cross shape (Figure 2A, Figure 3A). By contrast, ∼50% of *rsu-1**(RNAi)* and *rsu-1**(**tm6690**)* vm1 muscles were irregular in shape and harbored blister-like protrusions (Figure 3A and B). These results indicate that RSU-1 is required for cell morphology control.

Cell membrane blebbing was previously reported in MCF10A cells with overactivated R-Ras (Ada-Nguema *et al.* 2006). Therefore, we wonder whether the blebs observed here in *rsu-1**(**tm6690**)* might be caused by aberrant levels of Ras^LET-60^ activity. To address this possibility, we examined the shape of vm1 muscles after downregulating and upregulating Ras^LET-60^ activity. We downregulated Ras^LET-60^ activity by using *let-60**(RNAi)*, whereas for upregulation of Ras^LET-60^ activity, we used the strain *let-60**(**n1046**)* and *let-60**(**n1700**)*, in which these alleles in the chromosome IV encode a Ras^LET-60^ dominant mutant that is constitutively active (Han *et al.* 1990; Beitel *et al.* 1990). In *let-60**(**n1046**)* and *let-60**(**n1700**)* worms expressing P*egl-15*::GFP, ∼48% of vm1 muscles showed blister-like protrusions (Figure 3A and B), phenocopied *rsu-1**(**tm6690**)*. According to this observation, we then speculate that Ras^LET-60^ in the *rsu-1**(**tm6690**)* worms might be overactivated. Therefore, knockdown Ras^LET-60^ in *rsu-1**(**tm6690**)* should suppress the membrane blebbing. To test this, we treated *rsu-1**(**tm6690**)* or *rsu-1**(RNAi)* worms with *let-60**(RNAi)*. The blister-like protrusions were absent in l*et-60**(RNAi)*;*rsu-1**(**tm6690**)* adult worms or decreased to 8% in *let-60**(RNAi);**rsu-1**(RNAi)* adult worms (Figure 3 and S2). The penetrance of *let-60**(RNAi)* was confirmed by evaluating the number of vulva in *let-60**(**n1046**);**let-60**(RNAi)*, in which the number of vulva in *let-60**(**n1046**)* were significantly reduced (Fig. S3). Collectively, our data indicate that RSU-1 regulates Ras^LET-60^ to maintain the cell shape of vm1 muscles.

### RSU-1 promotes actin-bundle organization

**Figure 4:**
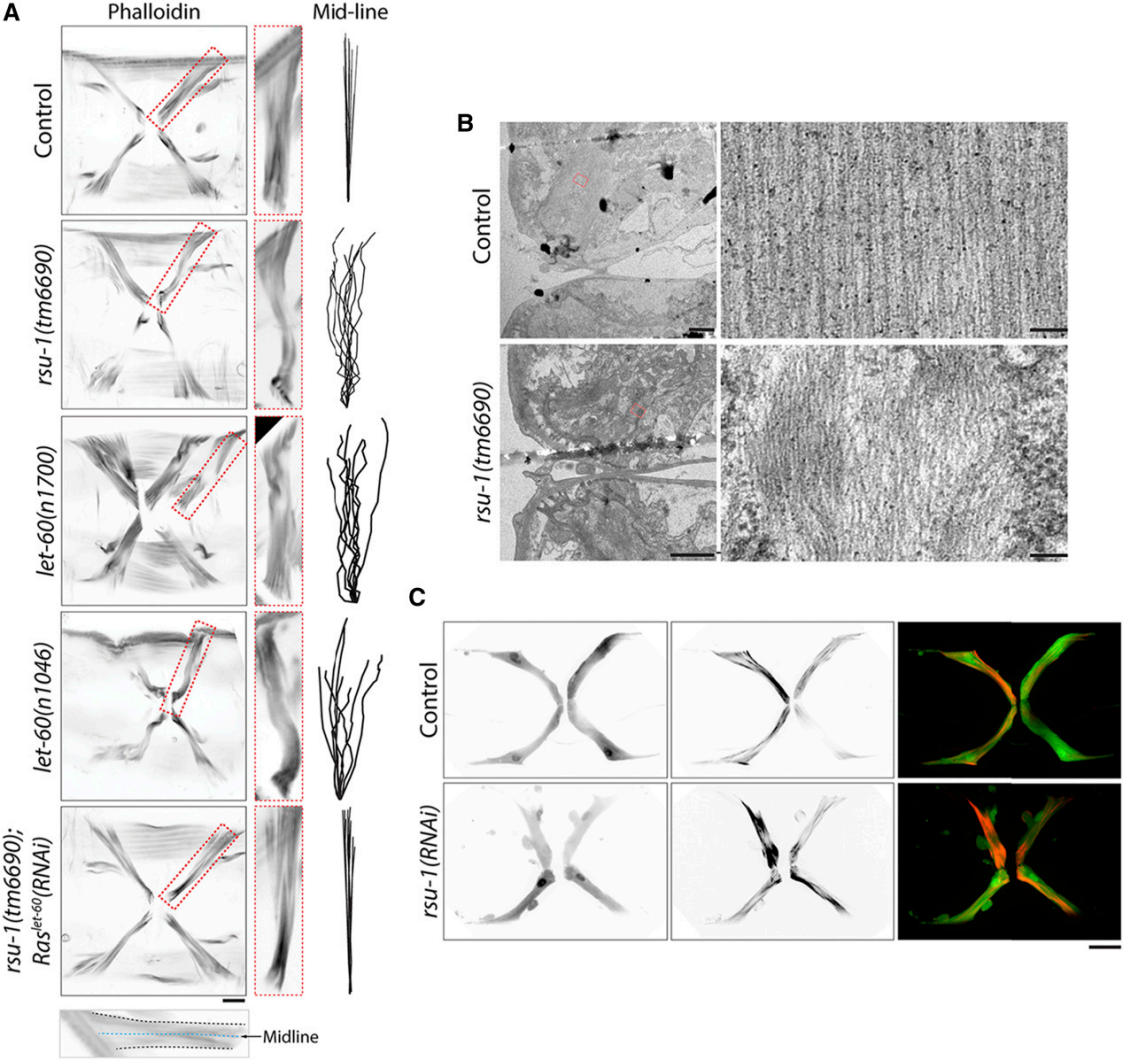
RSU-1 promotes actin-bundle organization by inhibiting Ras activity in vulval muscle. (A) Representative confocal images of control, *rsu-1**(tm6690)*, *let-60**(**n1700**)*, *let-60**(**n1046**)*, and *rsu-1**(tm6690);Ras**^let-60^**(RNAi)* muscle cells stained with iFlour 555-phalloidin. Red box: zoomed-in view of a single vm1 muscle cell. Actin-bundle midline in vm1 muscles was analyzed as shown at the bottom; each actin-bundle midline is displayed on the right. Scale bar, 10 μm. (B) Representative TEM images of vm1 vulval muscle in control and *rsu-1**(**tm6690**)*. Scale bar, 10 and 5 μm. (C) Representative confocal images of P*egl-15*::GFP;LifeAct::mKate and P*egl-15*::GFP;LifeAct::mKate;*rsu-1**(RNAi)*. Scale bar, 10 μm.

Blebs are generally generated in cells with abnormal cortical cytoskeleton (Gagliardi *et al.* 2015; Caswell and Zech 2018), therefore we further speculate that RSU-1 stabilizes the cytoskeleton in vm1 muscles to prevent deformation. To test this, we fixed adult worms and stained actin filaments with iFlour 555-phalloidin for actin bundle analysis. In control worms, most of the actin bundles in vm1 muscles were straight and parallel (Figure 4A). By contrast, curved actin bundles were observed in *rsu-1**(**tm6690**)* vm1 muscles, *let-60**(**n1700**)* vm1 muscles, and *let-60**(**n1046**)* vm1 muscles (Figure 4A). Notably, in agreement with the results of vm1 muscle-cell morphological analysis, the disorganization of actin bundles was suppressed in *let-60**(RNAi);**rsu-1**(**tm6690**)* vm1 muscles. We also examined the actin bundles in fixed *rsu-1**(**tm6690**)* by using transmission electron microscopy (TEM): whereas straight and parallel actin filaments were detected in control vm1 muscles, curved actin filaments with irregular empty spaces between actin filaments were observed in *rsu-1**(**tm6690**)* vm1 muscles (Figure 4B). More importantly, in live-worm confocal imaging, the disorganization of actin bundles and membrane blebbing were also observed in the *rsu-1**(RNAi)* vm1 muscles expressing LifeAct::mKate and P*egl-15*::GFP, which were used for (respectively) labeling actin filaments and visualizing the morphology of vm1 muscles (Figure 4C). These live-imaging data verified the reliability of the data from the analysis of fixed samples stained with phalloidin. Collectively, our findings demonstrate that RSU-1 regulates actin-bundle organization.

### RSU-1 regulates the subcellular localization of α-actinin^ATN-1^

**Figure 5:**
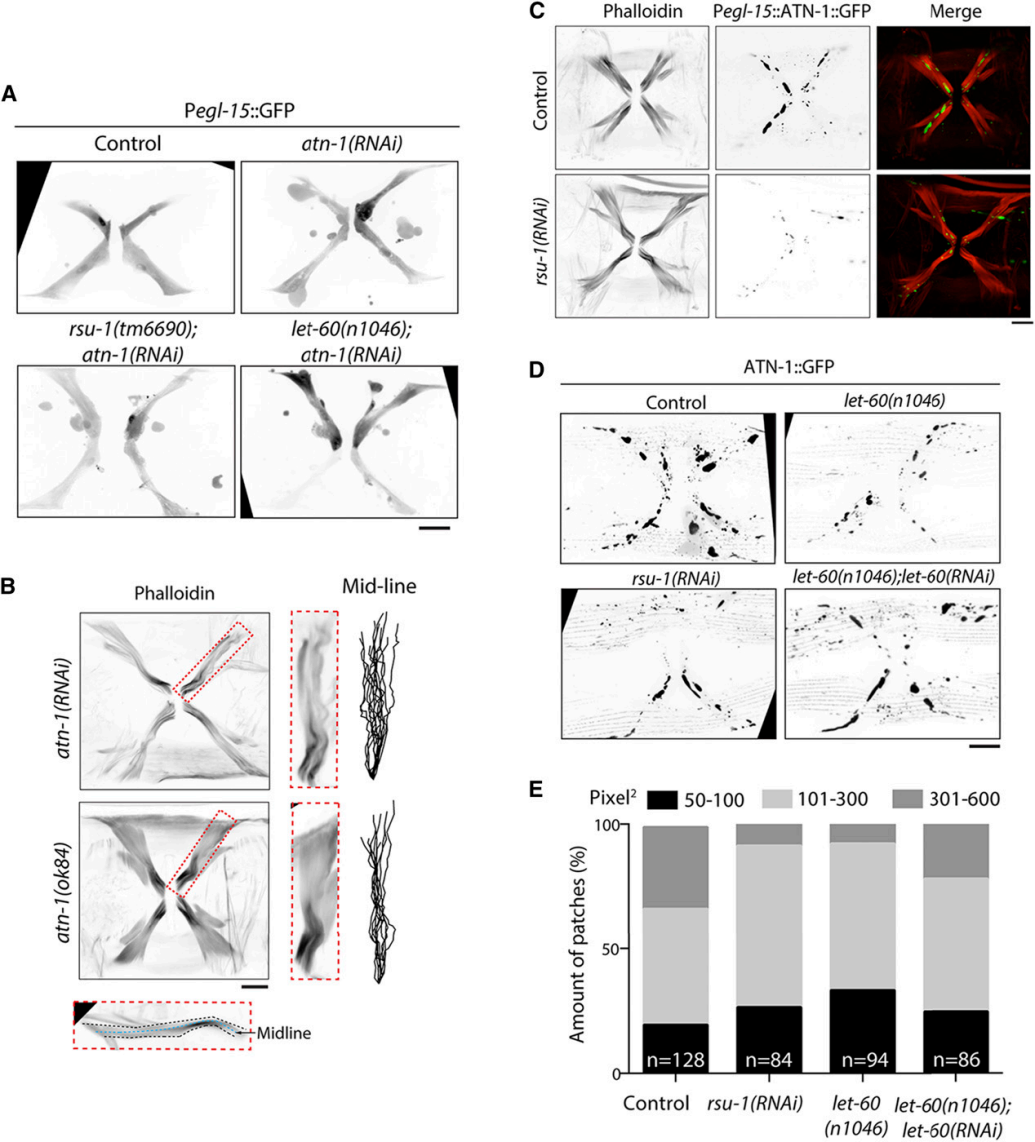
RSU-1 regulates α-actinin^ATN-1^ subcellular localization. (A) Representative confocal images of P*egl-15*::GFP in control, *atn-1**(RNAi)*, *rsu-1**(tm6690);**atn-1**(RNAi)*, and *let-60**(**n1046**);**atn-1**(RNAi)* muscle cells. Scale bar, 10 μm. (B) Representative confocal images of P*egl-15*::α-actinin^ATN-1^::GFP muscle cells in *atn-1**(RNAi)* and *atn-1**(**ok84**)* stained with iFlour 555-phalloidin. Scale bar, 10 μm. (C) Representative confocal images of P*egl-15*::α-actinin^ATN-1^::GFP and P*egl-15*::α-actinin^ATN-1^::GFP*;**rsu-1**(RNAi)* cells stained with iFlour 555-phalloidin. Scale bar, 10 μm. (D) Representative confocal images of P*egl-15*::α-actinin^ATN-1^::GFP, P*egl-15*::α-actinin^ATN-1^::GFP;*rsu-1*(*RNAi*), P*egl-15*::α-actinin^ATN-1^::GFP;*let-60**(**n1046**)*, and P*egl-15*::α-actinin^ATN-1^::GFP;*let-60**(**n1046**);**let-60**(RNAi)* samples. Scale bar, 10 μm. (E) Size of actinin patches in control, P*egl-15*::α-actinin^ATN-1^::GFP;*rsu-1*(*RNAi*), P*egl-15*::α-actinin^ATN-1^::GFP;*let-60**(**n1046**)*, and P*egl-15*::α-actinin^ATN-1^::GFP;*let-60**(**n1046**);**let-60**(RNAi)* vm1 muscles.

Actin bundles are stabilized by the interaction between many cross-linking proteins with actin filaments, forming a higher-order actin filaments structures in cells (Bartles 2000; Pollard 2016). To investigate how RSU-1 regulates actin bundling, we examined the cellular morphology of vm1 muscles in the depletion of actin-bundling proteins. After screening of several candidates by RNAi (Table S3), we observed that loss of α-actinin^ATN-1^ leads to membrane blebbing (Figure 5A). Furthermore, the blister-like protrusions were detected in both *rsu-1**(**tm6690**);**atn-1**(RNAi)* and *let-60**(**n1046**);**atn-1**(RNAi)* vm1 muscles (Figure 5A). These results indicate that α-actinin^ATN-1^ is required to maintain proper cellular shape in vm1 muscle, and that the protein might function downstream of RSU-1 and Ras^LET-60^.

Lastly, we analyzed the structure of actin bundles in vm1 muscles. Curved actin bundles were observed in *atn-1**(RNAi)* and *atn-1**(**ok84**)* cells (Figure 5B), which resembled the phenotype in RSU-1 mutant vm1 muscles (Figure 4A). We also examined the subcellular localization of α-actinin^ATN-1^ by expressing vm1 tissue-specific α-actinin^ATN-1^::GFP, which revealed that α-actinin^ATN-1^::GFP patches of different sizes were formed and were localized at the interspace between the actin bundles in vm1 muscles (Figure 5C). Notably, these patches were fewer and smaller in *rsu-1**(RNAi)* or *let-60**(**n1046**)* vm1 muscle cells than control cells, while the patches in *let-60**(**n1046**);**let-60**(RNAi)* were similar to control cells (Figure 5D and E). These results demonstrated that RSU-1 regulates α-actinin^ATN-1^ subcellular localization through Ras^LET-60^.

### Fifth to Seventh LRR of RSU-1 is required to stabilize vm1 morphology

**Figure 6:**
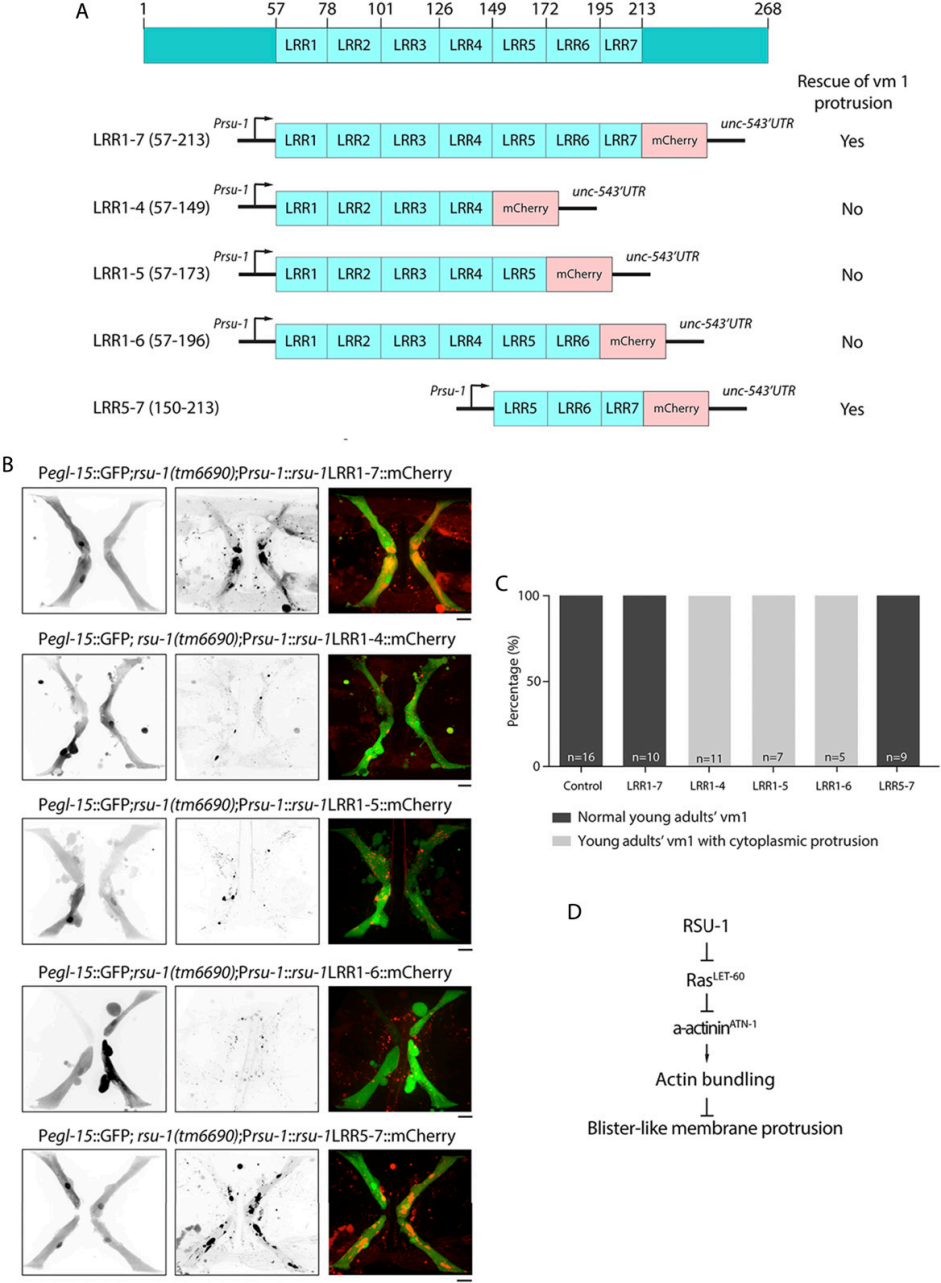
LRR5-7 domains of RSU-1 are required for vm1 morphology. (A) Schematic showing the truncated RSU-1-mCherry fusion proteins in *rsu-1**(**tm6690**)* mutants. The morphology of vm1 muscle cells in the rescue experiments was listed on the right. Yes: vm1 is normal; No: blister-like protrusion in vm1. (B) Representative confocal images of expressing truncated RSU-1(LRR1-7), (LRR1-4), (LRR1-5), (LRR1-6) or (LRR5-7) in P*egl-15*::GFP;*rsu-1**(**tm6690**)*. Scale bar, 10 μm. (C) Percentage of vm1 muscle cells showing cytoplasmic protrusions in (B). (D) Working model of RSU-1-mediated actin bundle organization. Our study suggested that RSU-1 suppresses Ras^LET-60^ to prevent the loss of subcellular localization of a-actinin^ATN-1^ in vm1 muscles, which consequencely prevent the formation of blister-like protrusion.

RSU-1 has seven LRR-containing domains, and they may form an arc or horseshoe shape, with the variable convex face and the parallel β-strands-containing concave face (Ng and Xavier 2011). We further investigated which region of RSU-1 is required for the regulation of α-actinin^ATN-1^ by extrachromosomal array-mediated rescue experiment. We expressed several truncated RSU-1-mCherry fusion proteins, driven by the RSU-1 promoter, in the *rsu-1**(**tm6690**)* worms, and then examined the morphology of vm1 muscle cells (Figure 6A). The LRR1-7::mCherry fusion protein formed large patches around the vulva, phenocopied the localization of wild-type RSU-1 (Figure 6B). However, the other truncated RSU-1-mCherry fusion proteins were diffused and formed small puncta in vm1 muscle cells (Figure 6B). More importantly, LRR1-7::mCherry and LRR5-7::mCherry suppressed blister-like protrusions in *rsu-1**(**tm6690**)*, whereas LRR1-4::mCherry, LRR1-5::mCherry, and LRR1-6::mCherry did not (Figure 6B & C). These data suggest that the fifth to seventh LRR-containing domain of RSU-1 is required for vm1 muscle cell morphology stabilization.

## Discussion

### RSU-1 functions independently of the focal adhesion pathway

Body-wall muscles are striated muscles that consist of multiple sarcomeres (Gieseler *et al.* 2018), whereas nonstriated smooth muscles, like vulval muscles in *C. elegans*, lack highly organized sarcomeres. The two major components of sarcomeres are M-lines and dense bodies, both of which are composed of several proteins associated with integrin, a transmembrane receptor that facilitates cell-extracellular matrix adhesion (Mackinnon *et al.* 2002; Lin *et al.* 2003). Previous work and our study have shown that RSU-1 is mainly expressed in body-wall muscles and colocalizes with Integrin^PAT-3^-localized structures, but RSU-1 is not required for the integrity of M-lines and dense bodies in body wall muscle (Fig. S4A & B) (Pierron *et al.* 2016). Concurrently, in vm1 muscles, our results also demonstrated that although the majority of RSU-1 is localized at focal adhesion sites, these sites remain intact in the loss of RSU-1 (Fig. S5A & B). Altogether, these findings suggest that RSU-1 may not function in the focal adhesion or it acts in parallel with an unknown protein in the downstream signaling of the focal adhesion pathway.

Intriguingly, in vm1 muscles, we identified blister-like protrusions containing defected actin bundles in *rsu-1**(RNAi)* and *rsu-1**(**tm6690**)* mutant worms (discussed below) but they were eliminated in the loss of Ras^LET-60^. Furthermore, we observed that RSU-1 was diffusely distributed in the cytoplasm of vm1 muscles. Therefore, based on the results of our genetic studies and subcellular-localization analysis, we propose that the cytosolic RSU-1 performs a cellular function that is independent of the integrin-mediated adhesion pathway in vm1 muscles. In human cells, RSU-1 was reported to regulate p38 activity in a PINCH^UNC-97^-independent manner (Gonzalez-Nieves *et al.* 2013), and was further reported to promote the viability of the *Drosophila* larva in a PINCH^UNC-97^-ILK^PAT-4^ interaction-defective mutant (Elias *et al.* 2012). Thus, it would be of interest to comprehensively investigate the molecular functions of RSU-1 in cellular processes.

### RSU-1 regulates the cellular structure of vm1 muscles

Cytoplasmic protrusions that perform specialized cellular functions, such as lamellipodia, filopodia (Mogilner and Rubinstein 2005), cytonemes and tunneling nanotubes (Buszczak *et al.* 2016), display specific cytoskeletal arrangements. However, dysfunctional cytoplasmic protrusions, named blebs or blister-like protrusions, have been observed in apoptotic cells (Sgonc and Gruber 1998), injured cells (Malorni *et al.* 1991), and tumor cells (Sahai and Marshall 2003). These blebs are generally generated by the actomyosin contraction that squeezes the cytosol outward at sites exhibiting local delamination of the cortical cytoskeleton (Caswell and Zech 2018). In this study, blister-like protrusions were observed in vm1 muscles lacking RSU-1 (Figure 3A) or α-actinin^ATN-1^ (Figure 5A). Unexpectedly, highly curved actin bundles, visualized by means of phalloidin staining or LifeAct::mKate labeling, were concomitantly detected in the cells under these conditions. Therefore, although we did not examine the dynamics of the cortical cytoskeleton, we suggest that these disorganized actin bundles could explain the bleb formation observed in vm1 muscles. Moreover, the blister-like protrusions were mainly detected in adult worms and not in L4-stage worms (Fig. S6). This further suggests that RSU-1 might not regulate the early development of vulval muscle but might contribute to the stabilization of actin bundles and thus enable the cells to withstand the mechanical stresses generated by muscle contraction during body locomotion and egg-laying.

### RSU-1 promotes actin-bundle stabilization

Actin filaments assemble into higher-order crosslinked bundles and thereby regulate cellular shape and cell adhesion (Bartles 2000). The actin-bundling process is mediated by actin-bundling proteins, including vinculin, fascin (Winkelman *et al.* 2016), and α-actinin (Bartles 2000). In human cells, actin-bundling proteins are closely colocalized with actin bundles (Hoffmann *et al.* 2014), although these proteins might form aggregates in certain specific cell types, such as in transformed rat kidney cells (Stickel and Wang 1987). Here, we also showed that in vm1 muscles, α-actinin colocalizes with rod-shaped patches located between well-defined actin bundles (Figure 5C), and further that α-actinin is required for maintaining the cellular structure and actin-bundle organization in vm1 muscles (Figure 5A and B). These results suggest that the α-actinin-containing patches hold actin bundles tightly together to prevent actin-bundle distortion.

Interestingly, our results also demonstrated that the size of the α-actinin-positive patches were regulated by RSU-1 in vm1 muscles (Figure 5D and E). Moreover, the loss of RSU-1 and that of α-actinin produced the same phenotypes in vm1 muscles. This indicates that α-actinin might act downstream of RSU-1. Our findings further demonstrated that Ras^LET-60^ overactivation leads to bleb formation and actin-bundle distortion, and, more importantly, that RSU-1 inhibits Ras^LET-60^ (Figure 3A & B, Figure 4A). Therefore, we propose that α-actinin^ATN-1^ acts as a downstream effector in the RSU-1-mediated actin-bundle stabilization pathway. In D*rosophila*, RSU-1 contributes to PINCH stability, and perhaps regulate actin network through the integrin-mediated signaling pathway during Drosophila development (Kadrmas *et al.* 2004). However, in vm1 muscle cells, RSU-1 is more likely to regulate actin filament organization in an integrin-dependent manner.

In summary, our results demonstrated that RSU-1 depletion, Ras^LET-60^ overactivation, and α-actinin^ATN-1^ depletion lead to the formation of blister-like protrusions in vm1 muscles. Furthermore, the results of confocal imaging and TEM analyses showed that the actin-bundle integrity in vm1 muscles was compromised under these three conditions. Consequently, egg-laying ability was diminished in the worms in which vm1 muscles harbored the protrusions. Our genetic studies further revealed that RSU-1 regulates actin-bundle stabilization by inhibiting α-actinin^ATN-1^ subcellular localization through Ras^LET-60^ (Figure 6D). Although the mechanism by which RSU-1 inhibits Ras^LET-60^ remains unclear, our data in the rescue experiments indicate that the fifth to seventh LRR-containing domains (LRR5-7) are critical for the inhibition. Therefore, the binding of RSU-1 and Ras^LET-60^ need to investigate further. Nevertheless, the interaction of a LRR protein, FLI-1, with Ras was reported in *C. elegans* during embryonic cytokinesis and germline development (Deng *et al.* 2007; Lu *et al.* 2008). In addition, another LRR protein, SOC-2 also directly interact with Ras and Raf to positively modulate Ras pathway in *C. elegans* (Sieburth *et al.* 1998; Selfors *et al.* 1998). Besides, previous studies have shown that α-actinin^ATN-1^ is activated by PIP2 binding and forms an antiparallel dimer (Ribeiro *et al.* 2014), and that Ras^LET-60^ activates PI3K to phosphorylate PIP2 into PIP3 (Kölsch *et al.* 2008). Therefore, we cannot rule out the possibility that the inhibition might not be simply due to a direct protein-protein interaction: RSU-1 might inhibit Ras^LET-60^-dependent activation of PIP2 phosphorylation, and this, in turn, might promote the activation of α-actinin^ATN-1^ for actin-bundle stabilization. This poorly understood mechanism underlying actin organization warrants further investigation.
